# Who bears the cost of ‘informal mhealth’? Health-workers’ mobile phone practices and associated political-moral economies of care in Ghana and Malawi

**DOI:** 10.1093/heapol/czw095

**Published:** 2016-07-31

**Authors:** Kate Hampshire, Gina Porter, Simon Mariwah, Alister Munthali, Elsbeth Robson, Samuel Asiedu Owusu, Albert Abane, James Milner

**Affiliations:** 1 Department of Anthropology, Durham University, Durham DH1 3LE, UK; 2 Department of Geography and Regional Planning, University of Cape Coast, Ghana,; 3 Centre for Social Research, University of Malawi; 4 Department of Geography, Environment and Earth Sciences, University of Hull, UK; 5 Department of Population and Health, University of Cape Coast, Ghana; † Deceased author

**Keywords:** Care work, community health-workers, mobile phones, moral economy, political economy, Sub-Saharan Africa, task shifting

## Abstract

Africa’s recent communications ‘revolution’ has generated optimism that using mobile phones for health (mhealth) can help bridge healthcare gaps, particularly for rural, hard-to-reach populations. However, while scale-up of mhealth pilots remains limited, health-workers across the continent possess mobile phones. This article draws on interviews from Ghana and Malawi to ask whether/how health-workers are using their phones *informally* and with what consequences. Health-workers were found to use personal mobile phones for a wide range of purposes: obtaining help in emergencies; communicating with patients/colleagues; facilitating community-based care, patient monitoring and medication adherence; obtaining clinical advice/information and managing logistics. However, the costs were being borne by the health-workers themselves, particularly by those at the lower echelons, in rural communities, often on minimal stipends/salaries, who are required to ‘care’ even at substantial personal cost. Although there is significant potential for ‘informal mhealth’ to improve (rural) healthcare, there is a risk that the associated moral and political economies of care will reinforce existing socioeconomic and geographic inequalities.

Key Messages

- In the absence of large-scale mhealth programmes, health-workers in Ghana and Malawi are using their own mobile phones to bridge healthcare gaps, particularly for rural, hard-to-reach populations.
- Health-workers use their own mobile phones for many purposes: to obtain help in emergencies, communicate with patients/colleagues, obtain clinical advice/information and manage logistics.
- However, the costs of this ‘informal mhealth’ are being borne by the health-workers themselves, particularly by low-paid community health-workers serving impoverished rural settlements.
- Serious consideration should be given to the implicit task shifting and cost shifting that informal mhealth may entail, to maximize healthcare benefits without placing excessive demands on health-workers.

## Introduction

### mhealth in Africa—potential and practice

Africa’s communications ‘revolution’ has generated optimism that ‘mobile health’ (mhealth)^1^ can help bridge persistent healthcare gaps. Mobile phone penetration rates across the continent are approaching 75% (685 million subscriptions by end of 2015; ITU 2015). Across Africa, mhealth schemes have been implemented to facilitate communication and information exchange (WHO 2011; Bastawrous and Armstrong 2013; Aranda-Jan *et al.* 2014; Folaranmi 2014). Applications include SMS appointment/medication reminders (Lester *et al.* 2010; Bigna *et al.* 2014); advice/information for community case management and referral (Campbell *et al.* 2014; Schuttner *et al.* 2014; Tumusiime *et al.* 2014); supporting routine maternal and infant care (Ngabo *et al.* 2012; Little *et al.* 2013; Crawford *et al.* 2014; Velez *et al.* 2014); drug supply and stock management (Campbell *et al.* 2014); public health surveillance (Chaiyachati *et al.* 2013; Brinkel *et al.* 2014; Madon *et al.* 2014) and staff training, support and monitoring (Zurovac *et al.* 2012).

Although most initiatives to date have been small-scale pilots, with limited evaluation and scale-up, some success stories are emerging. Particularly promising are schemes that facilitate the work of community health-workers (CHWs) serving rural or other hard-to-reach populations (Mahmud *et al.* 2010; Kallander *et al.* 2013; Little *et al.* 2013; Zurovac *et al.* 2013; Campbell *et al.* 2014; Schuttner *et al.* 2014; Tumusiime *et al.* 2014; Velez *et al.* 2014). Such initiatives resonate closely with a global health policy agenda that has, since the 1978 Alma Ata ‘*Health for All*’ Declaration, positioned CHWs as the linchpin of primary care in settings with high disease burdens and health-worker shortages (Haines *et al.* 2007; WHO 2008; Lewin *et al.* 2010; Singh and Sachs 2013).

mhealth is also associated with another global health policy preoccupation: cost reduction and ‘efficiency’. Based on a systematic review across Sub-Saharan Africa, Betjeman *et al.* (2013:1) assert that ‘mhealth can improve and *reduce the cost* of patient monitoring, medication adherence and healthcare worker communication, especially in rural areas’ (emphasis added; see also Folaranmi 2014). For example, a pilot scheme in Malawi that provided 75 CHWs with mobile phones reported saving over 2000 hours of worker time plus US$3000 fuel costs (Mahmud *et al.* 2010); likewise Odendaal and Lewin (2014) noted that cell phones could improve South African CHWs’ efficiency by avoiding long, unnecessary or fruitless walks to patients’ homes. Studies from India (Rodrigues *et al.* 2014) and Uganda (Chang *et al.* 2013a) reported mobile messaging to be a *cost-effective* means of improving anti-retroviral adherence while, in Kenya, mobile phone-based reminder systems have proven to be an ‘*effective and inexpensive*’ way to improve health-workers’ adherence to malaria case-management guidelines (Zurovac *et al.* 2012).

To summarize, most commentators agree that mhealth has the *potential* to improve healthcare in low-resource settings in a ‘cost-effective’ and ‘efficient’ way, especially by supporting the work of CHWs; however, this has not yet translated into large-scale investment (Folaranmi 2014; Chib *et al.* 2015). In the meantime, we know that most health-workers across Africa (in common with the wider population) possess mobile phones (Chang *et al.* 2013b; Zurovac *et al.* 2013). What we don’t know is whether—and how—they are using those phones *informally* to support healthcare delivery and with what consequences. Drawing on interviews in Malawi and Ghana, this article begins to address this important gap.

### Primary healthcare and mhealth in Ghana and Malawi

Malawi has over 10 000 health surveillance assistants (HSAs) —around 30% of the total public-sector health workforce—who form the backbone of primary healthcare. HSAs act as primary contact points between communities and health facilities; in practice they are often the only trained health-workers serving rural communities (Malawi Ministry of Health 2012; APC 2014; Smith *et al.* 2014). HSAs undergo 12 weeks’ post–secondary-school training and receive a salary of around US$100/month plus further training allowances. Their responsibilities include providing routine maternal and child healthcare; treating/referring cases of acute infectious disease; providing home-based care for patients with HIV, TB and other chronic conditions and delivering community health promotion/education. HSAs also supervise the work of village health committees (VHCs): elected community representatives charged with health promotion outreach and liaising with HSAs over local healthcare delivery (APC 2014). VHC members undergo a basic 5-day training programme; as ‘volunteers’, they receive no payment or allowances (Ibid).

In Ghana, by contrast, community healthcare has hitherto been provided principally by salaried nurses with a minimum of 2 years post-secondary training. This is now changing: the Government recently published a roadmap for training and deploying 28 000 CHWs (‘*a lower level cadre of health professionals who can be trained quickly to deliver preventive and curative services at the household level*’) by 2019 (Ghana Ministry of Health 2014; see also Singh and Sachs 2013). However, at the time of fieldwork, community health nurses (CHNs) were still the main providers of primary care at community level, with a similar set of responsibilities to Malawian HSAs.

Both Malawi and Ghana are actively promoting mhealth within primary care; each country has over 30 active mhealth projects operating, ranging in scale, reach and remit, from CHW-operated registration/data collection tools, to SMS appointment reminders and targeted patient messaging, to applications for monitoring essential medicine supplies (GSMA 2014a,b). However, in both countries, lack of coordinated partnership with government agencies and heavy reliance on international donor funding remain significant challenges for scale-up (Ibid). Roll-out in Malawi is further hindered by relatively low rates of phone ownership/access: only 36% of the population is estimated to have access to a mobile phone (compared with 85% in Ghana)^2^ and phone running costs in Malawi are among the highest in Africa, consuming on average 20% of users’ monthly incomes (GSMA 2014b).

## Methods

The data presented here come from a large inter-disciplinary study, conducted in Ghana, Malawi and South Africa, to establish the impacts of mobile phones on young people’s lives. Fieldwork was conducted (2012–2015) in 24 study sites across the three countries: one high-density urban, one peri-urban and two rural (one with basic services, one without), in each of two agro-ecological zones per country (Ghana: coastal savannah and central forest belt; Malawi: Lilongwe plains and Blantyre/Shire Highlands; South Africa: Eastern Cape and Gauteng/North-West provinces). All the urban sites were situated close to public hospitals and other health facilities, peri-urban sites usually had more basic (mostly nurse-led) clinics, while formal healthcare provision in rural sites was much sparser.

In each field-site, in-depth interviews were conducted with young people, parents and community key informants, including health-workers.^3^ The health-worker interviews—which form the empirical basis of this article—were designed to elicit views/experiences regarding the impact of mobile phones on healthcare, especially for young people. Unfortunately, logistical difficulties/delays prevented us from completing the health-worker interviews in South Africa; this article therefore draws on those from Ghana (*N* = 16) and Malawi (*N* = 18) only.

Research teams visited primary healthcare facilities in all sites where these were available. In the urban sites with multiple facilities, we selected those most often frequented by study participants; in rural settlements with no clinic, local volunteer groups (including VHCs in Malawi) were contacted. The research team explained the purpose and procedures of the research to the in-charge medical officer (or equivalent), and consent was sought to interview health-workers who were present/available during the team’s visit^4^; individual consent was also sought separately from each interviewee. Interviews were conducted usually in a private room in the clinic (if available) or another location where privacy could be ensured. Mindful of health-workers’ busy schedules and associated time/opportunity costs, interviews were kept to a maximum of about 45 min and scheduled at each interviewee’s convenience.

All health-worker interviews were conducted one-to-one, mostly in local languages, by an in-country academic collaborator or trained research assistant. Individual interviewing helped ensure confidentiality (a particular concern given professional hierarchies) and enabled participants to reflect in detail on their experiences. Interviews were semi-structured, starting with general background questions about work/responsibilities before asking more specifically about the role of mobile phones. All interviewees appeared to speak freely and openly; most had a lot to say on the subject matter with relatively little prompting required.

Interviewers took detailed hand-written notes during each interview, which were typed as soon as possible afterwards,^5^ and translated into English (leaving key/untranslatable terms in the original language). The resulting transcripts were read several times and annotated for emergent themes by (KH) in close consultation with in-country collaborators. Analysis was based on the principles of grounded theory, whereby theoretical insights emerge from the data rather than being pre-established (Strauss and Corbin 1998). Preliminary codes and themes were derived through a first close reading of transcripts; these were used to generate working hypotheses, which were then ‘tested’ and refined through further interviews and re-reading of transcripts.

Two important caveats should be noted. First, the sample of health-workers is small and not statistically representative. Sampling was opportunistic, as described earlier, although efforts were made to ensure that a range of health-worker cadres were interviewed in each location. In the event, the Malawi sample consisted predominantly of lower-paid HSAs and unpaid volunteers (mostly men) while, in Ghana, there were more qualified nurses (mostly women) working at community level, reflecting health systems differences outlined earlier: Table 1. Although not reflected in our sample, it should be noted that women form a majority of lower-paid/unpaid health-workers worldwide (Jenkins 2011); a point we to return later.

**Table 1:** Healthcare personnel interviewed in Malawi and Ghana

Job title/role	Numbers interviewed				
	Urban/Peri-urban		Rural		TOTALS
	Women	Men	Women	Men	
**(a) Malawi**					
Private Doctor	0	1	0	0	**1**
NGO Clinic Administrator	0	1	0	0	**1**
Medical Assistant	1	0	0	0	**1**
Midwife Nurse	1	0	0	0	**1**
Community Health Nurse (CHN)	1	0	0	0	**1**
Health Surveillance Assistant (HSA)	1	3	0	3	**7**
Village Health Committee (VHC) Members	0	3	2	1	**6**
**Malawi Total**	**4**	**8**	**2**	**4**	**18**

**(b) Ghana**					
Clinic-based Nurse (General/Registered/enrolled)	1	4	2	0	**5**
Community Health Nurse (CHN)	4	0	4	0	**8**
Health Assistant (HA)	0	0	1	0	**1**
**Ghana Total**	**5**	**4**	**7**	**0**	**16**

**Grand total**	**9**	**12**	**9**	**4**	**34**

Second, we had not intended to collect data specifically on the phone-related costs and moral conundrums that we discuss later. Instead, this was an *emergent* theme in the Malawi interviews (conducted first), which we then pursued more systematically in the ensuing Ghana interviews. This iterative process, whereby preliminary analysis informs subsequent data collection, constitutes the basis of grounded theory. Consequently, the same questions were not asked systematically of all interviewees and the study should thus be seen as exploratory. Nonetheless, we believe that the insights generated are important and suggest a significant research and policy gap.

## Results

### Health-workers’ mobile phone use

Only two interviewees had access to a workplace (mobile) phone: the Malawian private doctor and the Medical Assistant; the latter was part of an mhealth paediatric triage pilot and was restricted to that purpose. Two interviewees in Ghana reported having previously used workplace phones which were no longer working. However, all but three interviewees owned a mobile phone and reported using them extensively in their work; the three that didn’t (all Malawian VHC volunteers) often borrowed one from family/friends. Mobile phones were used for a variety of purposes, including most of those documented in the ‘formal mhealth’ literature: obtaining help in emergencies, communicating with patients/colleagues, obtaining clinical advice/information and managing logistics.

#### (a) Emergency use

All interviewees emphasized the value of mobile phones in medical emergencies, particularly in remote areas. Several had received emergency calls from distressed patients; many others had used their personal phones to request urgent advice from colleagues or to call an ambulance (or other emergency transport). All six VHC volunteers in Malawi had made calls on behalf of community members who had been taken seriously ill—or gone into labour—in the night while, for some health centre staff, making emergency calls had become routine:

Every day we have emergencies at the health centre. […] Today, for example, I called an ambulance for a patient who has cardiac problem. […] Another call was for a gynaecological patient with bleeding from a possible spontaneous abortion. (Malawi, female Medical Assistant, peri-urban)

#### (b) Community-based care and communication with patients

Besides emergency use, all health-workers—especially community-based ones—reported using personal cell phones for more routine communication with patients. These two accounts are typical:

Since TB is very critical in the first 2–8 weeks, we monitor the patients very closely during this period […] If a patient is on treatment and does not come for the next medication, I phone to find out if they are having any challenges. (Malawi, male HSA, urban)

For most of our diabetes and hypertensive patients, I have their phone numbers and they have my number as well. I call to monitor them and encourage them to take their medications and remind them to come for check-ups every month. (Ghana, female CHN, rural)

In turn, patients regularly call health-workers, who generally gave out their phone numbers freely. Interviewees’ estimates of how many patients had their personal numbers ranged from 10 to more than 40. Most reported receiving frequent calls from patients seeking reassurance, advice and sometimes emotional support (e.g. queries about how to take medication, worries about side effects or anxiety about test results). Again, these accounts are illustrative:

One woman, who came for [contraceptive implant] took my number. When she does not understand anything, she calls me for clarification. Another man came with twins for injections. The next day, he called because the children had some swelling. I told him that the swelling was a normal side effect so he should not worry, and I advised him to use an ice block and the swelling will reduce. (Ghana, female CHN, rural)

Some patients take my number to call in cases of emergency or if they need some advice. I had a patient—a married woman who tested HIV positive. […] She was afraid to reveal the news to her husband. I gave her my number so that she could call me before she told her husband. (Malawi, female CHN, urban)

#### (c) Communicating with colleagues

In addition to patients’ numbers, interviewees reported having between 10 and 100 colleagues’ contacts stored on their phones, which were used regularly to share information with peers, give instructions/information to subordinates and report problems or request advice from managers or supervisors. The following accounts illustrate these three different kinds of communication:

Sometimes you are on duty and realise that a patient is not doing so well, so you have to call a doctor. Sometimes it is a matter of life and death. […] The telephone exchange in the hospital is not working, so you have to pick your [mobile] phone and call the doctor’s number. […] When you are on night duty it is very crucial—you may lose the patient. (Ghana, male clinic-based nurse, urban)

We call our colleagues at other health facilities to inform them about patients we are referring details of their diagnosis and medication. […] Before we go for an outreach programme, we call colleagues to discuss preparation and what time to arrive. Almost every day I phone my colleagues about work. (Ghana, female CHN, urban)

Most often I use my phone to call the community volunteers to keep them informed about our programmes, such as visiting the aged, weighing infants, seeing to pregnant women, or vaccinating children. […] I use my phone on daily basis because we are always working in these communities and so before going there we call to inform the volunteers. (Ghana, female CHN, rural)

The increased availability of internet-enabled (3G) phones, particularly in Ghana, enables communication with colleagues via online messaging and networking applications. Over one-half of interviewees in Ghana used WhatsApp for this purpose; for example:

I am on WhatsApp with all my colleagues at the ward so when I am off duty I still get all the information. It keeps me updated on new admissions, who has been discharged or who has passed away. (Ghana, male clinic-based nurse, peri-urban)

Phone-based Internet has even allowed some to extend their professional networks further afield, as the Malawian Medical Assistant explained

I was on Facebook and found a cardiac professor and sent him a friend request. I communicate with him about drugs for patients. […] I have about 7 or 8 other Facebook contacts who are medical professionals in Malawi and abroad. (Malawi, female Medical Assistant, peri-urban)

#### (d) Seeking information

3G phones are also used by health-workers (again, mostly in Ghana) to search for other information online. As one Ghanaian CHN put it, ‘*itis not everything we are taught in school that we will always remember offhand. So you quickly browse the internet and get the required information*’. Interviewees reported having obtained information on various topics, from treatment of snake-bite to dosages of anti-malarial medicines; this account is typical:

At times you are working with a patient with a specific condition and you are confused as to what to do. But when you Google about the condition and find out the management, that will be helpful. (Ghana, male clinic-based nurse, urban)

#### (e) Drug supply management and other logistics

Finally, health-workers used their phones to manage daily work logistics. This is particularly important where inadequate/uncertain drug supplies are everyday challenges (Cameron *et al.* 2009). In Malawi, one HSA said he regularly calls the health centre when supplies of paracetamol, eye ointment or malaria medication are running low, while a nurse reported having called the Ministry of Health earlier that day to request an emergency delivery of anti-retrovirals. Similar situations were reported in Ghana, for example:

Sometimes I call other facilities to check for vaccines when we do not have adequate stock […] I can borrow some and replace it later. We even call the District office for assistance when other health facilities do not have enough stock. (Ghana, female CHN, urban)

When there is shortage of drugs, we put it on WhatsApp so that colleagues will inform us if they have excess for us to borrow. Some indicate which drugs they have run out of so that we will avoid referring patients there. (Ghana, male clinic-based nurse, peri-urban)

In summary, all health-workers and volunteers interviewed (even the three without their own phones) reported using personal cell phones regularly to facilitate many aspects of their work. Several claimed that cell phones could make (and in some cases, *had* made) the difference between life and death. Others said that mobile phones had enabled them to work more effectively and efficiently; e.g. checking patients’ or community volunteers’ availability before making a long journey. In other cases, phone calls *replaced* visits, as these two community nurses explained

Because of the rough roads, mobile phones help to reach the volunteers and patients in [distant] communities without necessarily going there. (Ghana, female CHN, rural)

But for mobile phone, our work would be difficult because for everything, be it assistance or enquiries, we would have to pick a taxi which would waste much time. (Ghana, female CHN, rural)

Many other interviewees echoed this point about saving time, emphasizing that mobile phones enabled them ‘*do things fast*’ and ‘*increase productivity*’. Most claimed that mobile phones were not only *useful*; they had become *indispensable*, particularly for those working in rural communities. One rural CHN in Ghana said, ‘*I use my phone almost every day in my work. You don’t have a choice*—*the only way to get the job done smoothly is to use my phone*’, while a rural health assistant (also in Ghana) put it even more dramatically: ‘*ifmy phone goes off, my whole world comes to an end because I cannot communicate with my colleagues and patients*’. This view is underlined by the accounts of health-workers without phones:

At first I was writing letters to communicate with fellow HSAs and VHC members […] Because I cover a big area, it was becoming a problem for me to walk to all those villages to inform them that I will visit. I could give messages to people attending the clinic, but it was taking long for the message to reach the intended village […] It was a big problem then to work as an HSA without a cell phone because not every message you pass to a patient reaches the village. […] I decided I had to have a cell phone to properly implement my duties, but the problem was that I did not have money. (Malawi, male HSA, rural)

I remember the times when work colleagues wanted me but could not communicate with me. Once, I needed to receive a drugs consignment at the Health Centre and get them into the drug store. Because they could not contact me, […] an HSA with no experience performed the duty. The danger is that he was not conversant with the logistics, book records, and could easily make mistakes. When I got back, I had to correct everything. (Malawi, male HSA, rural)

Even temporary phonelessness can be problematic. One Malawian HSA, whose phone was stolen in a public minibus, failed to see some TB patients because he hadn’t received an SMS. Another, whose phone had stopped working, missed an important briefing on cholera outbreak.

### Costs, challenges and motivations

Although mobile phones had become an integral part of our interviewees’ working lives, using them was not always straightforward. In both countries, poor or unreliable network was reported as a major issue in rural areas, despite improved coverage in recent years. Especially worrisome were emergency cases where, as one Ghanaian nurse put it, ‘*because of the poor network, it can result in a patient’s death*’. Another echoed this concern:

If there is an emergency and you can’t contact the medical director because of network problem, you do the little you can as a nurse but, if that doesn’t help, definitely you will lose the patient. (Ghana, female CHN, rural)

Network failure also led to meetings and other arrangements falling through, causing considerable frustration*.* One Ghanaian nurse recalled an occasion when a group of community volunteers had been kept waiting all afternoon because she could not communicate that her transport had broken down; others had made fruitless journeys to villages, only to find community members absent. Another rural health-worker in Ghana indicated a small hill that she would climb to obtain a phone signal but recalled an emergency case when general network failure had left her unable to call an ambulance. Similar problems arise when phones are out of battery charge, again especially in rural areas without mains electricity. One or two reported having purchased a spare battery or ‘power bank’ to mitigate this problem.

The other major challenge was phone credit (airtime), which came from the health-workers’ own pockets. Especially for those in lower-ranking (or unpaid) positions, this could represent a significant financial outlay. Many described it as a ‘burden’ and, as one Ghanaian CHN put it, ‘*it’snot always I can afford to buy airtime*’. In Malawi, where airtime is particularly expensive relative to incomes and living costs, several interviewees recalled occasions when lack of credit had prevented them making important calls. For example, one VHC Chairman reported sometimes failing to communicate with the HSA: the phone signal was often bad and, he said, ‘*if I do not have airtime, I may get discouraged and not call at all*’. In other cases, patient follow-up may be compromised or the financial burden shifted further to patients:

Airtime is becoming more expensive and we use our own resources to buy it. This is a big challenge. […] I have a wide area to cover. I may see over a 100 family planning clients and of these I may have to call 10–15 to follow up. If I can’t call all of them because of limited airtime, some may call us on their own […] or they may just disappear from the system. (Malawi, midwife nurse, urban)

Sometimes, if we don’t have airtime, we have to ask the patients, but not all patients have money for that or have their own cell phones. (Malawi, Medical Assistant)

Interviewees in Ghana generally managed to keep their phones in credit (a function probably of relatively higher salaries and lower phone costs), albeit sometimes at significant personal cost:

Sometimes you will have your phone with no credit but there is an emergency that requires calling a colleague urgently. In this case you’ll have no option than to use maybe the little money you have saved for something else to buy airtime. (Ghana, female CHN, rural)

I have to make other sacrifices just to afford the credit. Even if it is your last money, you have to sacrifice to buy credit so that the work can be done. (Ghana, female CHN, rural)

In addition to the financial cost, opportunities to buy credit may be limited, especially outside towns, as the Malawian medical Assistant explained

A month ago, I had a violent psychiatric patient here and I wanted transport for him to [Psychiatric Hospital]. I did not have any reliable drug for sedation. That day I didn’t have any credit in my phone. It was Sunday so the shops were closed. I had to sit here and watch the patient and give him short-acting drugs. […] It was very stressful and I was tired because whenever the patient woke up when the drugs wore off he became violent and I had to sedate him again. I tried to search for units at [trading centre] but they had run out of units and only one shop was open.

Finally, being permanently reachable by phone can carry significant *time* and *emotional* burdens. Almost all interviewees had become accustomed to receiving patients’ calls out of working hours, sometimes late at night:

Sometimes calls from patients can be a bother. When you close from work very tired and want to rest a little, patients will be calling. Sometimes they are asking for information that you have already given but they might have forgotten. […] Sometimes too they call even as late as midnight when you are tired and want to sleep. (Ghana, male clinic-based nurse, urban)

Sometimes, clients call at odd hours. Last year a patient called me at 5 am. I was deeply asleep when she called and said she was having menstrual pains and did not know what to do. (Ghana, female clinic-based nurse, urban)

Only one interviewee—a Malawian HSA—had stopped giving out his personal number to community members so freely, ‘*to avoid them bothering me with trivial issues or issues that are not related to health*’. However, his attempts were thwarted because other people continued to pass on his number.

Despite the costs involved, all our interviewees continued to use their personal phones and airtime for their work, even using money earmarked for other purposes. They expressed a strong moral imperative to alleviate patients’ suffering, even at personal cost. One Ghanaian CHN told us, ‘*asa human being you can’t just watch somebody dying just because you don’t use your mobile phone to call for assistance*’, an opinion widely shared by others:

I put myself in the shoes of the patient; he needs assistance and sometimes one call can save a life. So if you decide not to make the call or you become strict about how you use your mobile phone, you may lose a life. (Ghana, male clinic-based nurse, urban)

A more concrete example is provided by this Malawian HSA, who calls mothers as soon as their infants’ HIV test results come through. He explained

I use my own airtime because I feel sorry and guilty to delay the results to mothers. I don’t want to be the reason for delaying them knowing their babies’ results. (Malawi, male HSA, rural)

Many interviewees framed personal sacrifice in terms of a professional calling and/or religious duty. One Malawian HSA described health-workers as ‘*Good Samaritans*’, adding, ‘*professionally, you do not feel good to see a patient die or feeling great pain while you can help even with the little resources at your disposal*’. Likewise, a Ghanaian CHN said, ‘*wehave taken an oath to serve no matter the circumstance. […] We feel we owe a duty to serve people*’, while another told us, ‘*itis not good in the sight of God not to do [my job] well and so I sacrifice to do it well. […] What motivates me is the passion for the profession*’.

This *‘passion for the profession*’ was widely seen to distinguish a ‘good’ health-worker, marked by a willingness to use personal resources to help others:

You see, we don’t do this because of the money but the passion we have for the work. […] I know some nurses who will never use their own phones because they have no passion for the job. But, for some of us, it is the passion for the patients and the work that makes us continue. (Ghana, female CHN, urban)

If you don’t have the desire and passion for the work it makes you feel reluctant because you are using your own ways and means, so if the desire and passion is not there you can’t. It’s not everybody who will [use their own phone]. (Ghana, female CHN, rural)

The idea that *care work*, as a ‘charitable’ endeavour, should entail personal sacrifice was reproduced rhetorically both by government employers and health-workers themselves. One VHC Chairman asserted that ‘*the job is a voluntary one whereby we find our own means to get money for airtime to disseminate information to people*’, while an HSA told us, ‘*ourbosses at the ministry say that health work is charity work and we can use our own resources*’.

## Discussion

We reiterate the caveat that our findings derive from a small, opportunistic sample of health-workers in Malawi and Ghana and cannot be generalized more widely. However, the picture emerging is both encouraging and troubling. On the one hand, the potential for mobile phones to facilitate healthcare, especially in resource-poor and hard-to-reach settings, is clearly huge and it is remarkable how this is already being realized, in the absence of formal mhealth initiatives. A recent *Global Health: Science and Practice*editorial (2014:1) commented enthusiastically on health-workers’ ‘human ingenuity’ in this respect. On the other hand (at least in our study sites) the financial costs are largely being borne by health-workers themselves, and disproportionately by those working in rural communities, often on minimal stipends/salaries. As *de facto* ‘informal mhealth’ (Hampshire *et al.* 2015) becomes the norm, and both patients and providers come to rely on it, there is a serious risk of transferring the financial burden to those least able to afford it, who end up subsidizing healthcare delivery from their own pockets.

The fact that health-workers appear willing to do this resonates with a deeply rooted *moral* dimension to care work. Paying for airtime, charging and other phone maintenance costs is becoming part of a strong moral imperative to *care*, even at personal cost. The economies of care that ensue, which require low-paid (or unpaid) health-workers to forego financial reward and act selflessly, are thus profoundly *moral* ones. Rooted originally in the study of economic exchange in ‘pre-modern’, ‘peasant’ societies (Thompson 1971; Scott 1976), the concept of moral economies has been adopted more recently by some western feminist scholars as a useful way of thinking about relationships of care in which social values associated with gender identities may obscure structural and resource inequalities (see, e.g. McDowell *et al.*’s (2005) work on women’s unpaid childcare in UK). In an African context, anthropologists have used this concept to describe the values of selflessness and devotion—prominent in our interviewees’ narratives—expected of health-workers operating in socioeconomically disadvantaged communities (Prince 2012; Wendland 2012; Nading 2013; Swartz 2013).

But these moral economies of care are also, of course, deeply *political*. Governments and international donors have often been quick to adopt morally imbued arguments to justify minimal payment (or ‘compensation’) of CHWs (Maes and Kalafonos 2013). For example, new CHWs in Ethiopia are required to swear a public oath to ‘*put care recipients’ needs before their own*’ Maes (2014:107) and the Ethiopian Government justifies their non-payment by positioning CHWs as ‘*so valuable that they became “priceless” and therefore only remunerable with immaterial satisfaction*’ (Ibid:97). Likewise, Prince and Brown (2016) describe the implicit requirement for CHWs in East Africa to ‘*demonstrate a commitment to community development underlined by selflessness and the dedication of free labour*’—a message reinforced through bureaucratic techniques such as a Kenyan National Strategy document which cited ‘*respectability in the community and a* “*good heart*”’ among the selection criteria for prospective CHWs (Brown and Green 2015:71).

Such institutional rhetoric arguably then ‘*shap[es] CHWs’ [own] political subjectivities, motivations and capacities*’ (Maes 2014:108). For example, Glenton *et al.* (2010) found that CHWs in Nepal apparently resisted financial reward, which they believed would detract from the purity of altruistic motivation and undermine their social standing. Likewise, many of our interviewees were at pains to emphasize their ‘*passion*’ and desire to ‘*serve the people*’, reproducing official discourses that demarcate ‘good’/caring (selfless) from ‘bad’/uncaring (financially motivated) health-workers; a distinction that may be more rhetorical and symbolic than reflective of actual practice.

This moral framing of health-workers in general, and CHWs and volunteers in particular, is also closely associated with a wider neoliberal development discourse that has long promoted ‘community participation’ and voluntarism as routes to self-reliance. This ‘sustainability doctrine’ (Swidler and Watkins 2009) began to gain currency during the structural adjustment-enforced retrenchments of the 1980s/1990s, when public funding cuts shifted the financial burden for healthcare increasingly towards the private/voluntary sectors (Molyneux 2002; Jenkins 2009). More recently, it has re-emerged in the form of ‘task shifting’, promoted by the World Health Organization as ‘*the rational redistribution of tasks among health workforce teams […] from highly qualified health-workers to health-workers with shorter training and fewer qualifications’* as a ‘*pragmatic response to health workforce shortages*’ (WHO 2008:3; see also Zachariah *et al.* 2009).

However, as several commentators have observed, the burden of ‘community participation’ and ‘task shifting’ tends to fall disproportionately on the poorest: those required to ‘volunteer’ (or work for minimal stipends) are typically those at the bottom of healthcare hierarchies, and often women, who form the majority of CHWs worldwide (Molyneux 2002; Jenkins 2011:19; Brown 2013; Maes and Kalafonos 2013; Swartz 2013). Recently, Smith *et al.* (2014) noted that, in the context of ‘task shifting’, HSAs in Malawi were taking on many extra duties without adequate remuneration, leading to overload; informal mhealth may represent a further extension of this.

There is also a rural–urban dimension to consider. Although the literature highlights the particularly transformative potential of mhealth in rural communities with poor physical infrastructure, a corollary is that rural health-workers might bear the greatest burden of doing mhealth *informally*. Among our interviewees, it was those working in dispersed, rural communities who most often encountered situations that necessitated using their mobile phones. Rural CHWs, who are, on average, less well paid than their urban/clinic-based counterparts, risk spending a disproportionate share of their salaries subsidizing healthcare in this way, especially in countries like Malawi where phone costs remain high relative to incomes. It is also rural health-workers who bear the brunt of poor/unreliable network coverage and other infrastructural deficiencies (lack of battery-charging facilities or airtime retailers).

## Conclusion

Based on the—admittedly limited—data presented here, we would not disagree with the *Global Health*Editorial (2014) that called on the ‘global health community’ to encourage health-workers’ innovative use of mobile phones to bridge healthcare gaps, particularly in resource-poor, rural areas. Our interviewees are clearly doing this in Malawi and Ghana and the potential benefits are considerable.

However, we wish to sound a note of caution. First, it is important to ensure that quality of care is not compromised in the name of increased ‘efficiency’, e.g. through decreased face-to-face contact between health-workers and patients or community groups. Second, and more fundamentally, we must look carefully at *who* is bearing the costs, particularly where ‘informal mhealth’ becomes normalized, with concomitant expectations for health-workers and patients. Although the literature on formal mhealth emphasizes cost *saving*, *informal* mhealth may be more about cost *shifting*. Without critical analysis, that both engages with individual experiences and interrogates more profoundly the taken-for-granted neoliberal assumptions about ‘efficiency’ and public/private responsibility, there is a serious risk of perpetuating and reinforcing socioeconomic, geographical and perhaps gender inequalities. The strong moral imperative to *care* without financial reward renders CHWs especially vulnerable to economic exploitation. This is not just an issue of social justice: it has serious implications for the sustainability of programmes that rely on the motivation and commitment of CHWs who often struggle on daily basis to make ends meet (Akintola 2011; Maes *et al.* 2011).

Careful and critical social sciences research will be crucial in helping to address and mitigate these challenges. It would be pre-emptive to specify now what this might entail but, on a practical level, providing equipment (phones, solar chargers), airtime and/or allowances to CHWs seems an obvious step. Although such initiatives would require careful planning and monitoring, a small-scale trial along these lines in South Africa has been positively evaluated (Nxumalo *et al.* 2013). At a global level, serious consideration must be given to the implicit task shifting and cost shifting that informal mhealth may entail, and how to manage this to enhance capacity without compromising standards of care and the wellbeing of both health-workers and patients. As Ghana and other countries move to scale-up CHW deployment, getting this balance right must be a top priority.

## Ethical approval

- UK: Durham University Anthropology Ethical Review Board.
- Malawi: National Committee on Research in the Social Sciences and Humanities (NCRSH) at the National Commission for Science and Technology (NCST).

## Notes

1 mhealth refers to ‘the use of mobile and wireless technologies to support the achievement of healthcare objectives” (WHO, 2011:1).

2
http://www.itu.int/en/ITU-D/Statistics/Pages/default.aspx.

3 Approximately 80 interviews were conducted with young people (aged 9–25 years) per site, with smaller numbers of parent and key-informant interviews; a second phase entailed a questionnaire survey of 4500 young people across the 24 sites.

4 Research teams spent ∼2–3 weeks in each site, although not always consecutively.

5 We took a decision not to use recording equipment for both ethical reasons (concerns around potential sensitivities of the subject matter) and practical/logistical ones (risks of equipment failure/theft and time required to transcribe ∼1500 interviews in total).
